# The Low-density Lipoprotein Receptor-related Protein 6 Pathway in the Treatment of Intestinal Barrier Dysfunction Induced by Hypoxia and Intestinal Microbiota through the Wnt/β-catenin Pathway

**DOI:** 10.7150/ijbs.72283

**Published:** 2022-07-11

**Authors:** Zhihua Liu, Chao Li, Min Liu, Zhen Song, Mary Pat Moyer, Dan Su

**Affiliations:** 1 Department of Anorectal Surgery, the Fifth Affiliated Hospital of Guangzhou Medical University, Guangzhou, Guangdong, 510799, China.; 2 Key Laboratory of Biological Targeting Diagnosis, Therapy and Rehabilitation of Guangdong Higher Education Institutes, The Fifth Affiliated Hospital of Guangzhou Medical University.; 3 INCELL Corporation, San Antonio, Texas, 78249, USA.; 4 Department of Anorectal surgery. The Sixth Affiliated Hospital of Sun Yatsen University, Guangzhou 510665, China.

**Keywords:** gut microbiota, low-density lipoprotein receptor-related protein 6, endoplasmic-reticulum stress, beta-catenin, hypoxia, tight junction

## Abstract

Our study is to explore the key molecular of Low-density lipoprotein receptor-related protein 6 (LRP6) and the related Wnt/β-catenin pathway regulated by LRP6 during the intestinal barrier dysfunction. Colorectal protein profile analysis showed that LRP6 expression was decreased in dextran sulfate sodium (DSS)-induced colitis mice, and mice received fecal bacteria transplantation from stroke patients. Mice with intestinal hypoxia and intestinal epithelial cells cultured in hypoxia showed decreased expression of LRP6. Overexpression of LPR6 or its N-terminus rescued the Wnt/β-catenin signaling pathway which was inhibited by hypoxia and endoplasmic reticulum stress. In mice overexpressing of LRP6, the expression of β-catenin and DKK1 increased, Bcl2 decreased, and Bax increased. Mice with LRP6 knockout showed an opposite trend, and the expression of Claudin2, Occludin and ZO-1 decreased. Two drugs, curcumin and auranofin could alleviate intestinal barrier damage in DSS-induced colitis mice by targeting LRP-6. Therefore, gut microbiota dysbiosis and hypoxia can inhibit the LRP6 and Wnt/β-catenin pathway, and drugs targeting LRP6 can protect the intestinal barrier.

## Introduction

The role of the gut microbiota in intestinal barrier function has been increasingly studied 1-3. It is reported that intestinal barrier dysfunction is associated with the intestinal microbiota, which could also be treated by probiotics 4. The general therapeutic method for intestinal barrier dysfunction is using the probiotics 5-6. Also nowadays, fecal microbiota transplantation is developed for the protection of intestinal barrier. Some drugs also have a regulation on intestinal barrier and therapeutic effects on in intestinal barrier 7. Ischemia/reperfusion (I/R) injury is a model of intestinal barrier dysfunction 8-9, which could occur in multiple clinical settings, especially for patients in the intensive care unit 10-11. How I/R injury causes intestinal barrier dysfunction has been extensively studied 12, and oxidative stress, apoptosis 10, and autophagy were involved. As to the molecular pathway, the hypoxia-inducible factor-1α (HIF-1α) and Wnt/β-catenin pathways were mainly studied 10, 13.

The ischemia/reperfusion (I/R) injury is mainly caused by myocardial infarction, stroke, and peripheral vascular disease. I/R may lead to the production of various oxidant species, inflammatory factors, and nucleus and induce DNA injury, resulting in sepsis, tissue injury and organ failure finally. It is reported that I/R may lead to epithelial barrier dysfunction through hypoxia-inducible factor-1α in epithelial cell promotes expression of tight junction proteins 14. Cofilin could increase intestinal permeability via depolymerization of f-Actin during Hypoxia 15. Our previous study has also reported the relationship between epithelial dysfunction and gut microbiota 16. Recent years, fecal microbiota transplantation is developed for the protection of intestinal barrier. Furthermore, some drugs were also reported as methods to treat intestinal barrier dysfunction, such as Kaempferol, matrine, and sodium nitroprusside 17-19.

Wnt/β-catenin was reported to be an important pathway regulated by hypoxia, and HIF-1α extensively interacts with this pathway 20-21. During colorectal tumorigenesis in hypoxic conditions, HIF-1α competed with T-cell factor-4 for direct binding to β-catenin, which resulted in inhibition of Wnt pathway target genes and induction of HIF-1 target genes 22. In embryonic stem cells, HIF1α-β-catenin complexes could upregulate lymphoid enhancer-binding factor 1 and transcription factor 1, which might activate Wnt signaling 23. For osteoarthritis, the HIF1α-β-catenin interaction might be a negative regulator of Wnt signaling, thus reducing catabolism, while Wnt3a could promote β-catenin translocation into the nucleus in hypoxia 24. In cultured macrophages, HIF-1 mediated the increase in Wnt1 and β-catenin expression induced by hypoxia 13. HIF1α also repressed myogenesis through inhibition of canonical Wnt signaling 25. Therefore, the active or inhibitory effects of hypoxia on the Wnt/β-catenin pathway are still under debate. As for I/R injury, activation of the Wnt/β-catenin pathway might protect kidneys against I/R injury by attenuating apoptosis and inflammation of tubule epithelial cells 26, whereas the Wnt signaling pathway attenuated liver injury and improved the survival of rats induced by hepatic I/R 27. A recent study of intestinal I/R showed that Rg1 activated the Wnt/β-catenin signaling pathway both *in vivo* and *in vitro* and then reduced ROS and apoptosis 10. However, the Wnt/β-catenin pathway in gut barrier dysfunction has not been investigated. Our study aims to explore the Wnt/β-catenin pathway regulated by LRP6 during the intestinal barrier damage and find relative drugs targeting LPR6.

## Materials and methods

### Reagents and plasmids

Tunicamycin (TM), thapsigargin (TG), MG132, LiCl and chloroquine were purchased from Sigma-Aldrich. The reporter plasmid TOPFLASH and the LRP6 and LRP6deltN expression plasmids were bought from the Center for Cancer Systems Biology (CCSB)-Broad Lentiviral Expression Library.

### Cell culture and transfection

FHC and NCM460 cells were maintained in DMEM supplemented with 10% FBS, 100 μg/mL penicillin and 100 μg/mL streptomycin. FHC and NCM460 cells were transfected using Lipo 2000 (Lipofectamine™ 2000, Invitrogen) according to the manufacturer's instructions. After 24 h incubation at 37°C and 95% air and 5% CO_2_, the cells were subjected to the corresponding treatment and assay. To analyze the Wnt signaling pathway, cells were treated with Wnt3a-conditioned medium (WCM) or control medium (LCM) as preciously described 28.

### Mouse model

Male wild-type C57BL/6 mice at 5-7 weeks were obtained from the Model Animal Research Center of Nanjing University. The study protocol and experiments were conducted according to the guidelines of the Institutional Animal Care and Use Committee of Guangzhou Medical University and were approved by the Institutional Ethics Committee of Guangzhou Medical University. For the I/R model, 0.5 h of intestinal ischemia and 2 h reperfusion were performed 10. For the dextran sulfate sodium (DSS)-induced acute colitis model, DSS (40 kDa, Sigma-Aldrich, St. Louis, MO) in their drinking water in a 2% (w/v) solution (7 days) was used.

The LPR6 transgenic and knockout mouse lines were obtained as reported before 29-31. To get the intestinal-specific knockout or overexpression mice, targeting vector for overexpression or deletion mutation was established using the pBR322 vector which contains a neomycin resistance gene driven by the pGK promoter, flanked by FRT sites. Gene targeting process replaced the pre-miR sequences (391bp) flanked by loxP sites with neomycin resistance gene. Positive clones were injected into C57BL/6 blastocysts, >50% chimeric male mice were crossed to C57BL/6 females to generate flox+/+ or overexpression mice. Flox+/+ mice were intercrossed with Villin-Cre transgenic mice (J000664, Jackson laboratories) to remove the target gene in ECs to generated the intestinal-specific knockout mice. These mice were backcrossed to C57BL/6 mice for more than eight generations and homozygous for the relevant mutation. All of the mice were raised under specific pathogen-free conditions with filtered air, fed rodent chow ad libitum and allowed free access to water. Mice epithelial cells were performed by PCR, using genomic DNA isolated from the tail by the tissue DNA isolation kit (Biomiga, China).

For the mice (age 2 months) treated with drugs, the mice were treated with intraperitoneal administration of auranofin (10 mg/kg/day) or with auranofin (10 mg/kg/day), 5 days per week (Monday to Friday) for 2 weeks.

### Fecal 16S RNA sequencing

Fecal samples of stroke patients, mice with DSS-induced colitis, and ICU patients who were clinically diagnosed with intestinal barrier dysfunction and their corresponding controls were used in 16S RNA sequencing (Illumina HiSeq2500 PE250, GENE DENOVO).

### Fecal microbiota transplantation

C57BL/6 mice were given vancomycin (975 mg/kg, 1 day, Eli Lilly Japan K. K, Seishin Laboratories) and then polyethylene glycol (468 g/kg, 1 day, Shenzhen Vanward Pharmaceutical Co., Ltd.) to remove the original intestinal microbiota. Fecal microbiota transplantation was performed every other day three times using a gavage method.

### Microarray

Colon samples of mice with DSS-induced colitis/control and mice with fecal microbiota transplantation of stroke patients/healthy people were determined by microarray (L-Series Mouse Antibody Array L-308, Ray Biotech). The study design and protocols were reviewed and approved by the Human Research Review Committee of the Fifth Affiliated Hospital of Guangzhou Medical University.

### Hypoxic treatment

FHC and NCM460 cells were routinely cultured. Fresh medium was exchanged before treatment for varying amounts of time in a hypoxic incubator (Stem Cell Technologies), which was maintained an environment of 1% oxygen. Normoxic cells were cultured in 5% oxygen. All cells were supplemented with 5% CO2.

### Immunoprecipitation and Western blot analysis

After the hypoxic or TM&TG treatment, FHC and NCM460 cells were harvested by RIPA lysis buffer. β-catenin protein immunoprecipitated by 1 μg β-catenin antibody (BD Biosciences), cell lysates and antibodies were incubated in lysis buffer overnight on a shaker to allow the formation of protein-antibody complexes. Then, 30 μl Protein G-agarose beads were added to the corresponding sample for another 2 hours. Finally, the beads were collected by 1 min of centrifugation and washed four times with lysis buffer. SDS-PAGE was used, and immunoblotting the sample was performed with antibodies 32. The level of ubiquitination was determined by western blot 33.

### Real-time PCR analysis

Cells were harvested in 500 μl TRIzol Reagent (Ambion by Life Technologies), and the cDNA was synthesized by the manufacturer (PrimeScript™ RT reagent Kit with gDNA Eraser). TB Green Premix Ex Taq II (TaKaRa) and 7500 Real-Time PCR System (Applied Biosystems) were used. Relative changes in gene expression were normalized to β-actin RNA in the same cDNA sample 32. The primers used are shown in the Table S1.

### Immunofluorescence

Cells were fixed and labeled with anti-HA (Roche) and then stained with FITC (Invitrogen), and immunofluorescence images were acquired by an Olympus microscope (FV1200) 34. The image was analyzed by software (ImageJ).

### *In vivo* intestinal permeability assay

Mice were orally gavaged with fluorescein-isothiocyanate (FITC)-dextran (4 kDa, Sigma, 50 mg/100 g BW) 3 hours before sacrifice. Whole blood was obtained from the aorta abdominalis before overdose anesthesia. Plasma fluorescence was monitored using a spectrophotofluorometer (Varioskan lux, Thermo Scientific, USA) using excitation at 488 nm and emission at 528 nm. A standard curve was prepared using two fold dilutions of FITC-dextran in PBS 16.

### Statistical analysis

Statistical analysis was performed using the program SPSS for Windows Version 17.0 (SPSS, Chicago, IL, USA) and GraphPad Prism 5 software (San Diego, CA). Data are expressed as the mean ± SEM and were compared by analysis of variance (one-way ANOVA) with a Newman-Keuls post hoc correction for multiple comparisons or a t-test when appropriate. The differences between two groups were considered statistically significant if the p value was less than 0.05.

## Results

### The gut microbiota profile showed similarities between human and mice models with intestinal barrier dysfunction and the potential molecular of LRP6

**Figure 1:**
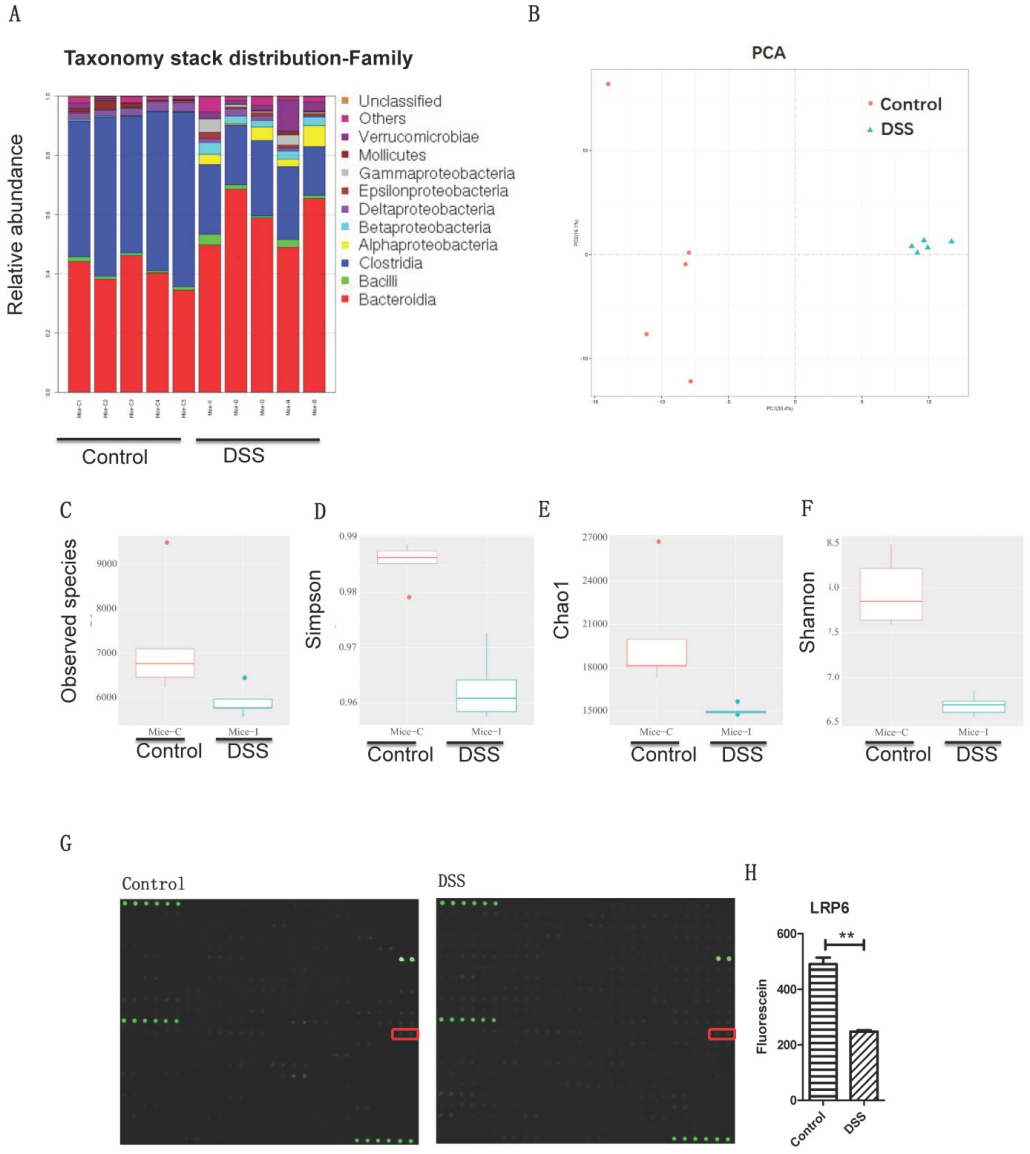
** Gut microbiota dysbiosis and intestinal barrier dysfunction were associated with lower LRP6 expression. (A)** Gut microbiota of controls and mice with DSS-induced colitis at the genus level (n=5 for each group). **(B)** PCA analysis of gut microbiota of controls and mice with DSS-induced colitis. **(C, D, E, F)** Statistic analysis of the corresponding difference of observed species, Simpson, Chao1, and Shannon. **(G)** Microarray analysis of the intestinal samples of fecal transplantation of healthy volunteers and of post-stroke patients. **(H)** Statistic analysis indicated the significant difference of LRP6. LRP6: low-density lipoprotein receptor-related protein 6; DSS; dextran sulfate sodium. Throughout, error bars represent the mean ± s.e.m. P values determined by Student's t-test, * vs control, P<0.05.

The results indicated that the microbiota profiles were different between DSS-induced colitis and the control (**Fig. 1A-1B**), ICU patients and healthy people (**Fig. S1A-S1B**), and post-stroke patients and healthy people (**Fig. S1C-S1D**) at the genus level. Interestingly, there are 51 common microbiota of the colitis mice, intensive care unit patients, and post-stroke patients (**Fig. 1C**): Coprococcus, Flexispira, Anaerotruncus, Proteus, Dorea, Moryella, Roseburia, Bacteroides, Desulfovibrio, Bifidobacterium, Lactobacillus, Vibrio, Oscillospira, Enterococcus, Anaerofustis, Dehalobacterium, Klebsiella, SMB53, Parabacteroides, Kurthia, Clostridium, Sutterella, Pseudomonas, cc_115, Prevotella, Persicobacter, Treponema, Brenneria, Eggerthella, Turicibacter, Coprobacillus, Coriobacterium, Acinetobacter, Kocuria, Adlercreutzia, Streptococcus, Lachnospira, Ruminococcus, Aggregatibacter, Paracoccus, Anaerostipes, Akkermansia, Brachybacterium, Ochrobactrum, Enhydrobacter, Erwinia, Salmonella, Faecalibacterium, Collinsella, Cupriavidus, and Thiobacter. These results indicate that gut dysbiosis microbiota may play a role in intestinal barrier damage.

To investigate the intestinal pathway induced by gut microbiota dysbiosis, fecal samples of stoke patients and healthy volunteers were transplanted to mice (**Fig. 1D**), and the colon profile was determined by microarray. Results indicated that LRP6 was significantly decreased in the colon samples of mice with DSS-induced colitis and mice with fecal transplantation of stroke patients compared with control groups (**Figure 1E**). Therefore, LRP6 was selected for further study of molecular mechanism during intestinal barrier dysfunction.

### LRP6 was inhibited by hypoxia and ER stress

**Figure 2:**
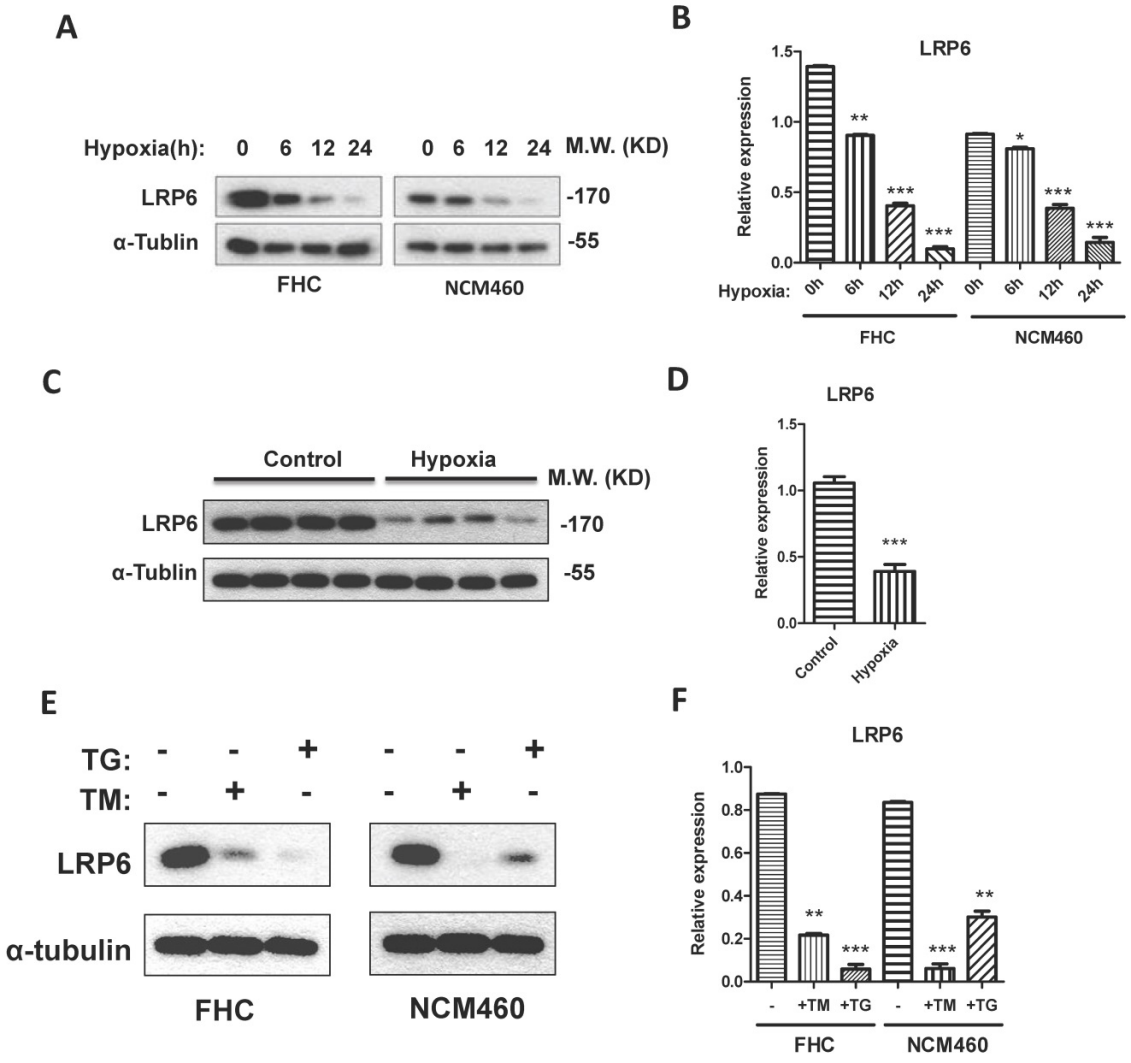
** LRP6 was inhibited by hypoxia through ER stress. (A, B)** Hypoxia could decrease the expression of LRP6 in intestinal cell lines. **(C, D)** Hypoxia could decrease the expression of LRP6 in mouse colon. **(E, F)** ER stress induced by TG and TM could decrease the expression of LRP6. Throughout, error bars represent the mean ± s.e.m. P values determined by Student's t-test. * vs control, P<0.05. LRP6: low-density lipoprotein receptor-related protein 6; TM; Tunicamycin, TG; thapsigargin.

To establish the model of hypoxia, the intestinal blood was blocked for 0.5 h for intestinal ischemia and reperfused for 2 h. Results showed that LRP6 was significantly decreased after the blood vessel was blocked (**Fig. 2C, 2D**). Interestingly, hypoxic culture of FHC and NCM460 cells could also inhibit the expression of LRP6 (**Fig. 2A-2D**). TM and TG were used to activate ER stress and investigate the effects of ER stress on LPR6. ER stress activated by TM or TG could downregulate the expression of LRP6 (**Fig. 2E-2F**).

### Hypoxia and ER stress downregulated expression of β-catenin and inhibited by overexpression of LRP6

**Figure 3:**
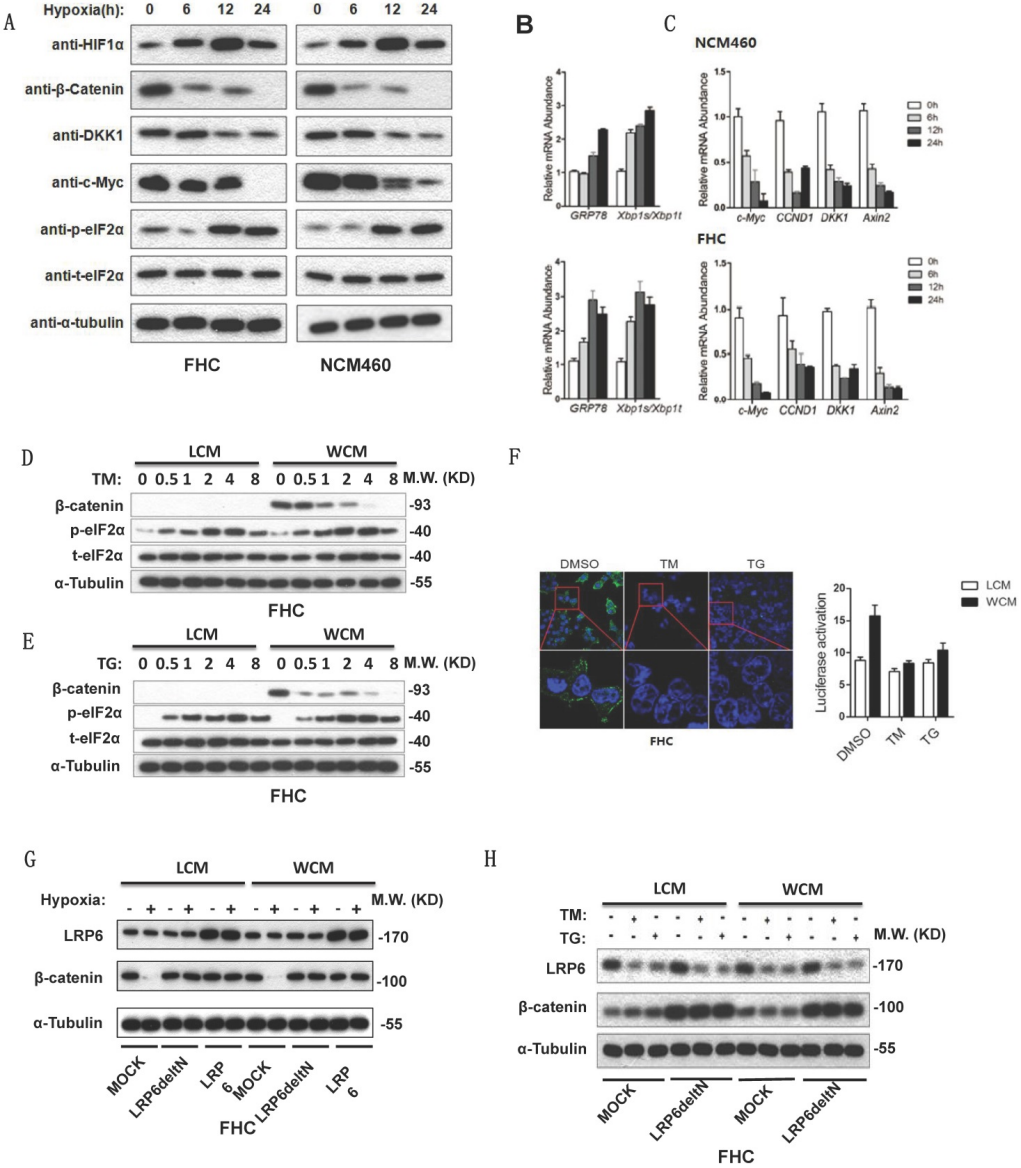
** Hypoxia and ER stress downregulated the expression of β-catenin which could be rescued by overexpression of LRP6. (A)** Protein expression levels of HIF1α, β-catenin and its downstream DKK1 and C-myc and the ER stress marker phosphorylated elF2α detected by Western blots using FHC and NCM460 cells. **(B)** mRNA level of the ER stress marker GRP78 and xbp1s/xbp1t of FHC and NCM460 cells treated by hypoxia (* vs control, P<0.05). **(C)** mRNA level of the Wnt/β-catenin target genes c-Myc, CCDN1, DKK1, and Axin2 of FHC and NCM460 cells treated by hypoxia (* vs control, P<0.05). **(D)** TG could increase the expression level of phosphorylated elF2α and decrease the expression level of β-catenin. **(E)** TM could increase the expression level of phosphorylated elF2α and decrease the expression level of β-catenin. **(F)** TG and TM could decrease the luciferase of the TOP FLASH assay for β-catenin (* vs control, P<0.05). The effect of TG and TM on the activity of TOP FLASH reporter is weak in LCM group, compared with the WCM group. **(G)** Hypoxia could also decrease the expression of β-catenin, which could be rescued by transfection of Delt N LRP6 or LRP6. **(H)** TG and TM could decrease the expression of β-catenin, which could be rescued by transfection of Delt N LRP6. HIF: hypoxia-inducible factor-1α; TM; Tunicamycin, TG; thapsigargin.

Hypoxia can activate ER stress and inhibit the Wnt/β-catenin pathway. Increased expression of phosphorylated elF2α, GRP78 and xbp1s/xbp1t indicated the activation of ER stress (**Fig. 3A-3B**). Decreased β-catenin and its target genes c-Myc, CCDN1, DKK1 and Axin2 inhibited the Wnt/β-catenin pathway (**Fig. 3A-3C**). TM or TG activated ER stress, then lowered the expression of β-catenin (**Fig. 3D-3E**). The TOP FLASH assay also indicated that ER stress could inhibit the activation of β-catenin (**Fig. 3F**). Furthermore, hypoxia decreased the expression of β-catenin, which could be rescued by transfection of Delt N LRP6 or LRP6 (**Fig. 3G**). Transfection of Delt N LRP6 rescued the expression of β-catenin, which was not impacted by TG and TM (**Fig. 3H**). Semi-quantitative analysis was shown in Fig. S2.

### Hypoxia-induced ER stress could be inhibited through ubiquitylation of β-catenin

**Figure 4:**
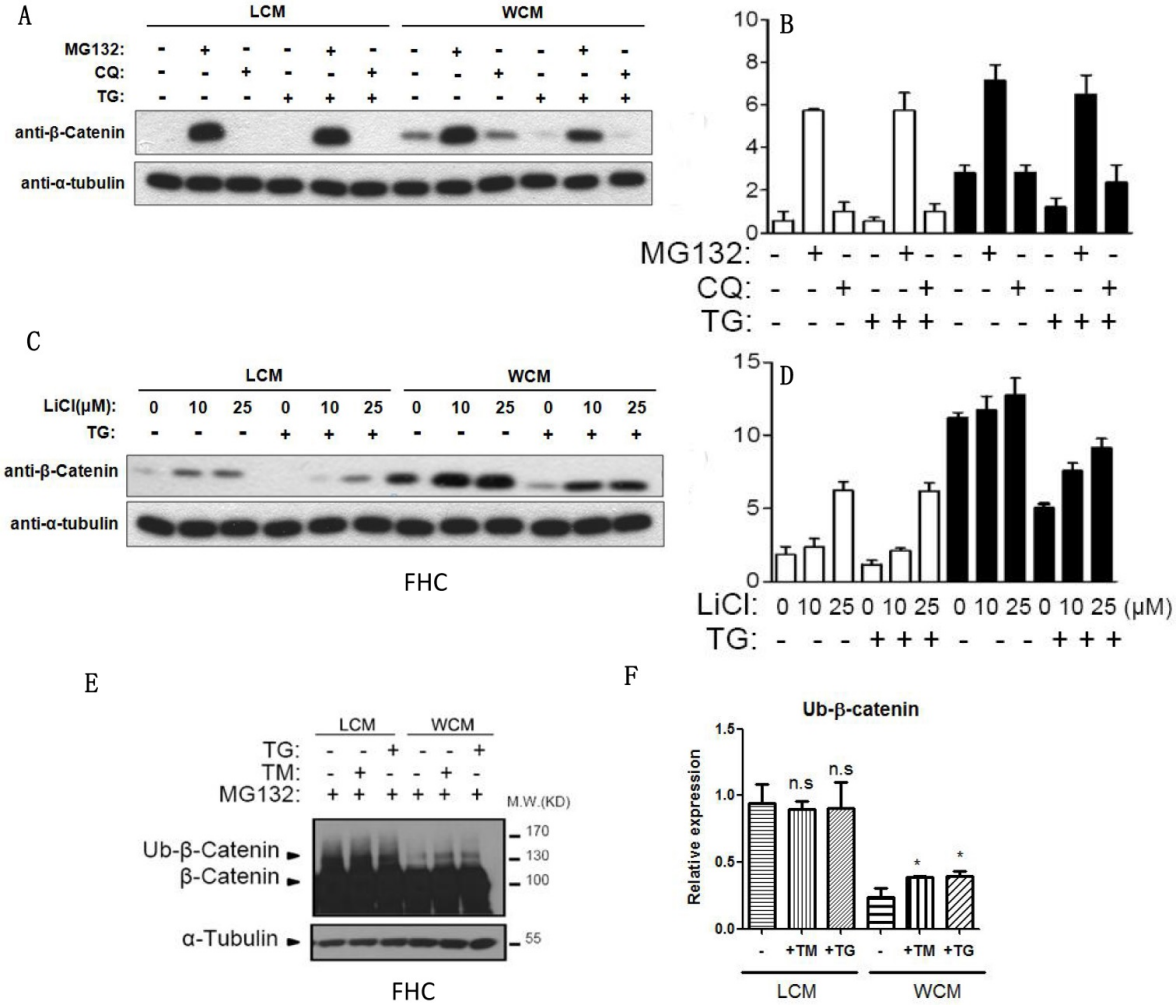
** ER stress could decrease the expression of β-catenin through ubiquitylation. (A, B)** The proteasome inhibitor MG132 could inhibit the decrease of β-catenin induced by TG. **(C, D)** LiCl inhibited the decrease of β-catenin that induced by TG. **(E, F)** The ubiquitylation of β-catenin could be induced by TG and TM, leading to the degradation of β-catenin after ER stress. Throughout, error bars represent the mean ± s.e.m. P values determined by Student's t-test. * vs control, P<0.05. TM; Tunicamycin, TG; thapsigargin; WCM: Wnt3a-conditioned medium; LCM: control medium.

When the proteasome inhibitor MG132 was added, β-catenin was exempted from the inhibition of chloroquine- and TG-induced ER stress (**Fig. 4A-4B**), and LiCl decelerated the decrease of β-catenin induced by ER stress (**Fig. 4C-4D**). The ubiquitylation of β-catenin could be induced by TG and TM, leading to the degradation of β-catenin (**Fig. 4E-4F**).

### LPR6 protected the intestinal barrier function

**Figure 5:**
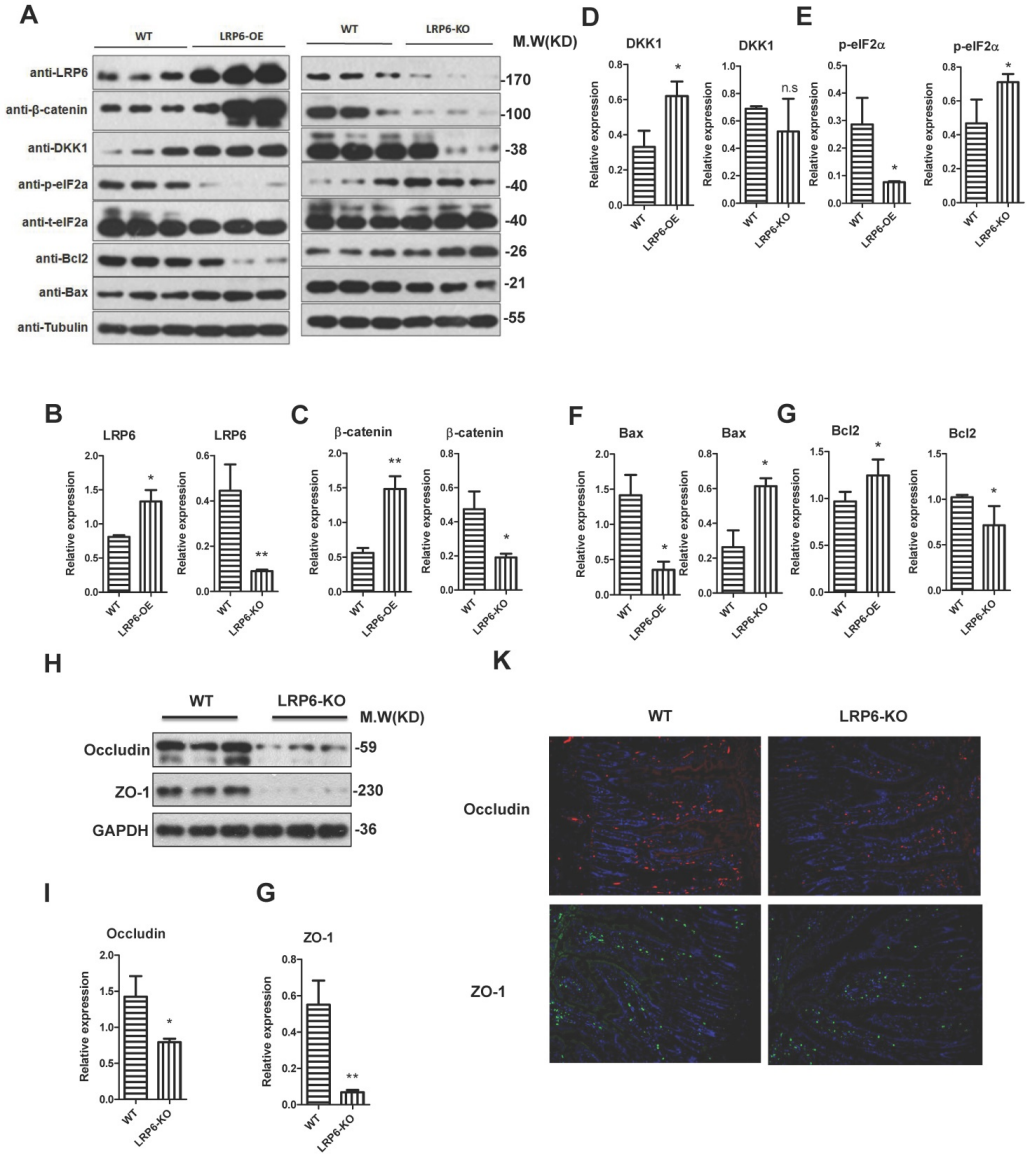
** Protective role of LPR6 on tight junction-associated proteins. (A-G)** The protein expression level of LRP6, β-catenin and its corresponding downstream molecule DKK1, ER stress-related p-eIF2α, proliferation marker Bcl2 and apoptosis marker Bax of wild type (WT), LRP6 overexpression (LRP6-OE) and LRP6 intestinal knockout (LRP6-KO) mice (n=3). **(H)** The protein expression level of occludin and ZO-1 of wild type and LRP6 intestinal knockout (LRP6-KO) mice (n=3) that detected by WB. **(I)** Semi-quantitative analysis of expression level of occludin that detected by WB. **(J)** Semi-quantitative analysis of expression level of ZO-1. **(K)** Immunofluorescent staining for the expression level of occludin and ZO-1. Throughout, error bars represent the mean ± s.e.m. * vs DSS, *P < 0.05; **P < 0.01; n.s., not significant; Student's t-test. WT: wild-type; LRP6: low-density lipoprotein receptor-related protein 6.

To further explore the biological role of LRP6, LRP6 intestinal knockout mice and LRP6 overexpression mice were constructed (**Fig. 5A**). The overexpression and deletion of LRP6 in mice was verified by PCR as show in Fig. 5B. The results indicated that the expression levels of β-catenin and its downstream DKK1 were significantly increased in LRP6 overexpression mice, while opposite results were found in LRP6 knockout mice (**Fig. 5A, 5C, 5D**). Similarly, ER stress-related p-eIF2α decreased in the LRP6 overexpression mice and increased in the LRP6 intestinal knockout mice (**Fig. 5A, 5E**). Furthermore, Bcl2 and Bax protein assays indicated that LRP6 could promote proliferation and inhibit apoptosis (**Fig. 5A, 5F, 5G**). Moreover, the expression levels of the tight junction-associated proteins occludin and ZO-1 were decreased in the LRP6 intestinal knockout mice, compared with the wild-type mice (**Fig. 5H, 5I, 5J**). These results indicated that LRP6 can protect intestinal barrier function through the Wnt/β-catenin pathway. The effect of LRP6 KO and overexpression on gut permeability was shown in Fig. S3 H.

### AU and CU can protect intestinal barrier function by activating LPR6

**Figure 6:**
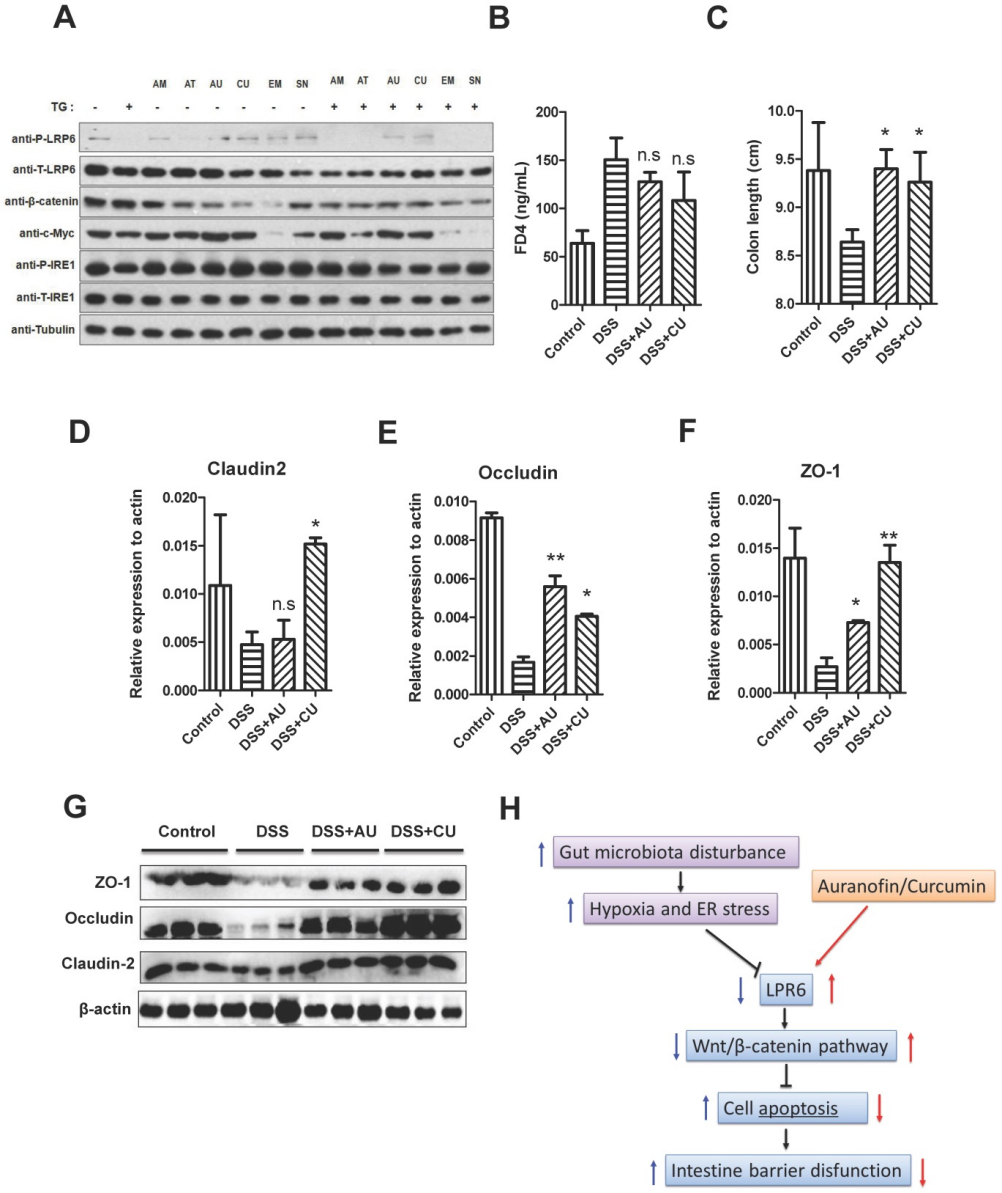
** AU and CU could protect intestinal barrier function by activating LPR6. (A)** Drug selection targeting the LRP6 pathway. **(B)** Intestinal barrier permeability of mice after treatment by DSS and DSS plus oral administration of AU and CU (n=6). **(C)** Colon length of mice after treatment by DSS with or without AU and CU (n=6). **(D)** Colon claudin2 mRNA of mice after treatment of DSS with or without AU and CU (n=6). **(E)** Colon occludin mRNA of mice after treatment by DSS with or without AU and CU (n=6). **(F)** Colon ZO-1 mRNA of mice after treatment by DSS with or without AU and CU (n=6). **(G)** claudin2, occludin and ZO-1 expression of mice colon after treatment by DSS with or without AU and CU (n=3). Throughout, error bars represent the mean ± s.e.m. * vs WT, *P < 0.05; **P < 0.01; n.s., not significant; by Student's t-test. LRP6: low-density lipoprotein receptor-related protein 6; AT: AT-7519; CU; curcumin; SN: SN-38; AU: auranofin; AM: amsacrine; and EM: emetine.

For the treatment of colitis, drugs including AT-7519, curcumin (CU), SN-38, auranofin (AU), amsacrine, and emetine were used for the selection of LRP6 activators. Although the TG was not significantly decrease the expression level of total LRP-6, it could lower the expression of phosphorylated LRP6. However, after the pretreatment of AU and CU, the expression of phosphorylated LRP6 was increased, indicating that AU and CU could rescue the phosphorylation of LRP6 after the addition of TG, then exerted the protective effects of intestinal barrier (**Fig. 6A**). Therefore, AU and CU were selected for the animal verification. Oral administration of AU and CU could protect DSS-induced colitis mice from intestinal barrier dysfunction through decreasing intestinal permeability and increasing the expression level of TJ proteins.

Intestinal barrier permeability of mice indicated that AU and CU could relieve the increase of intestinal permeability induced by DSS (Fig. 6B). Moreover, AU and CU could relieve the decrease of colon length induced by DSS (Fig. 6C). The intestinal TJ protein level of claudin2, occluding, and ZO-1 was increased after the pretreatment of AU or CU (Fig.6D-F). WB also showed the increase level of claudin2, occluding, and ZO-1 induced by AU or CU (Fig. 6G). Semi-quantitative analysis was shown in Fig. S3. The protective role of LPR6 in DSS-induced mice colitis was also verified by histological analysis of colon (Fig. S4).

## Discussion

In our present study, we first used three models of intestinal barrier dysfunction: post-stroke patients, intensive care unit patients, and mice with DSS-induced colitis 35. Stanley et al. analyzed the microbiota of lungs from sham-operated and post-stroke mice 12, whereas the intestinal microbiota analysis of post-stroke patients was first performed by our study. Furthermore, the signal transduction pathway after intestinal I/R injury was investigated using the FMT model of stroke patients 36. The microarray assay showed that LPR6 expression was significantly decreased in the gut microbiota dysbiosis and DSS-induced colitis mouse model group. Consistent with the previous study 10, the Wnt/β-catenin signaling pathway was inhibited in the intestinal I/R mouse model. Therefore, the signal transduction pathway of Wnt/β-catenin was further investigated in our study.

In embryonic stem cells and colorectal cancer cells, the Wnt/β-catenin pathway could either be inhibited or activated after the pretreatment of hopoxia 22-23. However, in the normal colonic cell line FHC and NCM460 cultured under hypoxic conditions, β-catenin was significantly decreased, and the target genes DKK1 and c-myc were decreased as well at both the mRNA and protein levels. Meanwhile, the expression levels of P-elF2α and the ER stress-related protein GRP78 were increased after the induction of hypoxia, indicating that ER stress was activated by hypoxia. Similar articles also reported that hypoxia might exert its biological function through the induction of ER stress. Verras et al. reported that microenvironmental stresses in solid tumors could inhibit the canonical Wnt/β-catenin signaling pathway with an underlying mechanism of hypoxia-induced ER stress that inhibits normal Wnt protein processing and secretion 37. Wang et al. found that *Panax quinquefolius* saponins reduce myocardial hypoxia-reoxygenation injury by inhibiting ER stress and the expression of GRP78 38. Our results confirmed that in normal cell lines of FHC and NCM460, hypoxia can inhibit Wnt/β-catenin through ER stress.

Therefore, TG and TM, two activators of ER stress, were used to verify the impaction of ER stress on the Wnt/β-catenin signal. Our results indicated that TG and TM could downregulate the expression of β-catenin and inhibit the activation of the Wnt/β-catenin signaling pathway in the normal cell lines FHC and NCM460. However, what happened to β-catenin? Does post-transcriptional degradation make sense? Or transcriptional inhibition has an important effect? Translocation of β-catenin was reported to be promoted by hypoxia 37. Interestingly, it is reported that the expression of β-catenin was not affected by O_2_ level, which might be related to the direct interaction of HIF1α and β-catenin 37. Our study further investigated the effects of post-transcriptional ubiquitylation of β-catenin and found that TG and TM could induce the ubiquitylation and degradation of β-catenin. This mechanism might be another signaling pathway of posttranscriptional regulation of β-catenin by ER stress, which was consistent with other scholars' findings in bone homeostasis, showing that phosphorylated β-catenin could be targeted for polyubiquitination and proteasomal destruction 39. Another study also showed that inhibiting β-catenin ubiquitylation could increase endogenous cytosolic β-catenin, which resulted in translocation of β-catenin into the nuclei and induced β-catenin-dependent gene expression 40.

How does ER stress impact the degradation of β-catenin? Canonical signaling was reported to be initiated by the binding of Wnt ligands to the dual receptor complex composed of frizzled and either LRP5 or LPR6 40, resulting in the inactivation of the multiprotein β-catenin-APC/Axin1 “destruction complex” 41 and then relieving β-catenin from its constitutive proteasomal degradation 39. However, when the receptor complex is not engaged, CK1 and GSK3 sequentially phosphorylate Axin-bound β-catenin at a series of regularly spaced N-terminal Ser/Thr residues, which could be ubiquitinated and targeted for rapid destruction by the proteasome 42. In our study, LRP6 was decreased by TG and TM treatment to activate ER stress, whereas delt N LRP6 could rescue the expression of LRP6 inhibited by hypoxia and ER stress. Sclerostin is a secreted glycoprotein that antagonizes Wnt signaling by binding to LRP5 and LRP6 39, 43, the monoclonal antibody of which might be used for an osteoanabolic drug target by targeting the Wnt/β-catenin signal pathway. Above all, we concluded that LRP6 could be a key molecule during the regulation of hypoxia to Wnt/β-catenin via ER stress.

**Figure 7:**
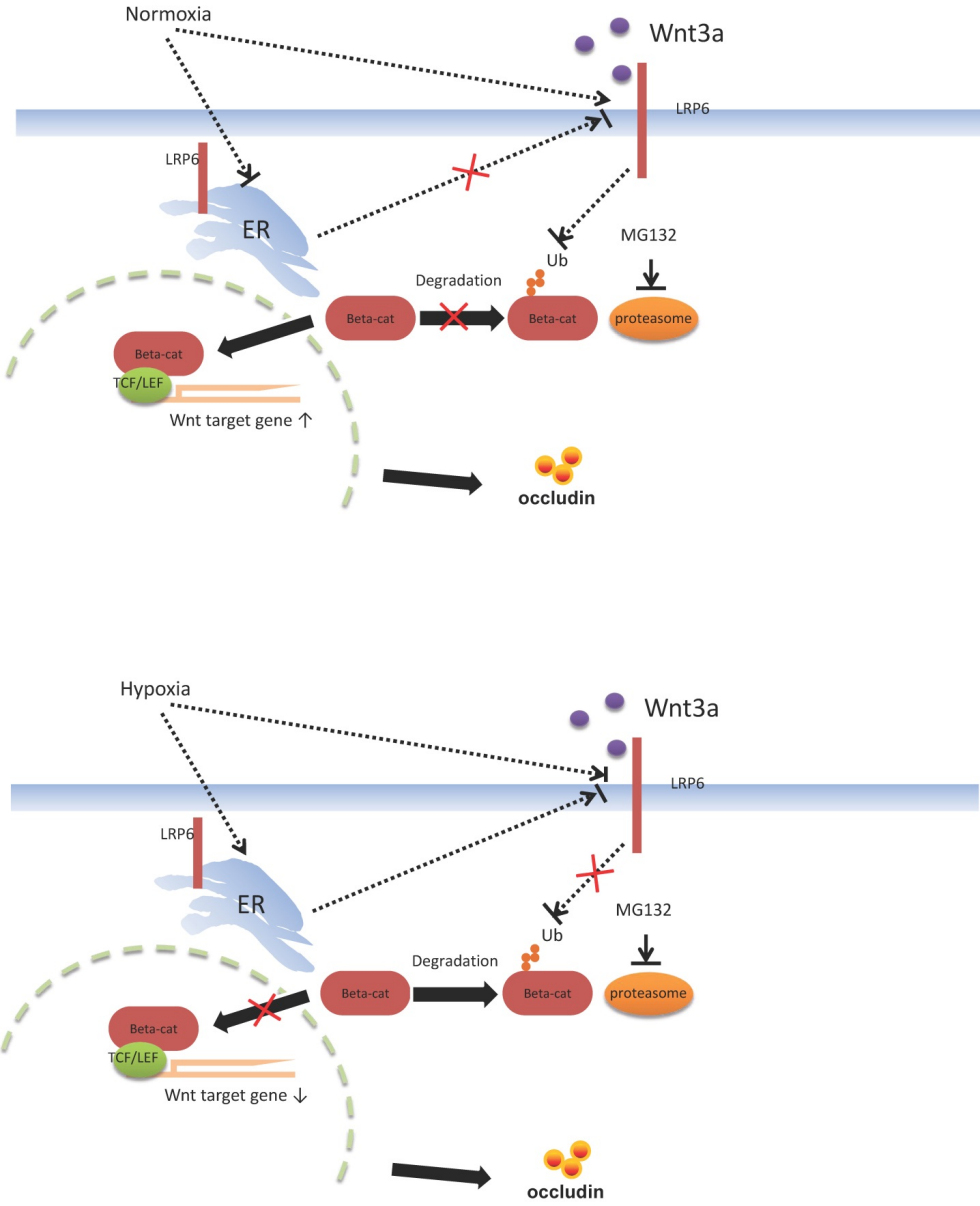
Proposed mechanism of intestinal barrier dysfunction which is induced by intestinal microbial dysbiosis and regulated by LRP6.

Functional verification of LRP6 was performed using LRP6 knockout or overexpression mice to verify the protective effects of LPR6 on TJ proteins and intestinal permeability. Oral administration of AU and CU, which target the activation of LRP6, could protect intestinal barrier dysfunction by increasing the expression level of TJ proteins and reducing the intestinal barrier permeability. CU is commonly used as coloring agent, food additive, and therapeutic drugs in intestinal inflammatory and other diseases 44. AU was normally used as the anti-cancer and anti-bacteria drugs 45-46. It is reported that CU was shown to improve intestinal barrier function through modulation of intracellular signaling and organization of tight junctions, including reducing IL1-induced activation of p-38 MAPK and subsequently increasing the expression of Myosin Light Chain Kinase involved in the phosphorylation of tight junction proteins and the ensuing disruption of their normal arrangement 47. Our results showed the therapeutic effects of AU and CU on intestinal barrier dysfunction, which may involve the regulating the LRP6 phosphorylation, and also indicated that AU and CU could be useful drugs for intestinal barrier dysfunction. Zhong et al. reported that conditionally deleted LRP6 in the mouse gut resulted in viable animals with apparently normal intestinal differentiation and function 48. Mice homozygous for deletion of both genes (*Lrp5^gut-/-^;Lrp6^gut-/-^*) died within one day of birth 48, with a progressive loss of cells, an absence of proliferation, reduction of a Wnt/β-catenin target, cyclin D1, and a premature differentiation of crypt stem/precursor cells 48. Several studies have reported the association between intestinal barrier function and intestinal microbiota 49-51. However, our study first emphasized the change in the intestinal barrier via the molecular mechanism of LRP6 via the pathway of Wnt/β-catenin. Furthermore, we also proposed possible therapeutic drugs target LRP-6 for intestinal barrier damage resulting from intestinal microbial dysbiosis (Fig. 7).

One limitation of our study is that the intestinal TJ proteins of clinical patients of intestinal barrier dysfunction were not investigated. Further study may need to investigate the clinical verification.

## Conclusion

Hypoxia could inhibit LRP6 via wnt/β-catenin pathway through intestinal microbiota, and drugs targeting LRP6 may help protect intestinal barrier function.

## Supplementary Material

Supplementary figures.
