# Supplementary material for: The Low-density Lipoprotein Receptor-related Protein 6 Pathway in the Treatment of Intestinal Barrier Dysfunction Induced by Hypoxia and Intestinal Microbiota through the Wnt/β-catenin Pathway
Source: Int J Biol Sci. 2022 Jul 11;18(11):4469–81. doi: 10.7150/ijbs.72283 (PMC9295061; doi:10.7150/ijbs.72283)

Figure S1 (A) Analysis of gut microbiota of ICU patients (n=10) and healthy volunteers (n=14) at the genus level; **(B)** PCA analysis of gut microbiota of ICU patients and healthy volunteers; **(C)** Analysis of gut microbiota of post-stroke patients (n=9) and healthy volunteers (n=9) at the genus level; **(D)** PCA analysis of gut microbiota of post-stroke patients and healthy volunteers. ICU: intensive care unit.

Figure S2 Semi-quantitative analysis of Figure3 D, E, G, H.

Figure S3 (A, G) Semi-quantitative analysis of of Figure 6A, G. (H) The effect of LRP6 KO and overexpression on gut permeability.

Figure S4 Histological analysis of colon in the animal models. The protective role of LPR6 in DSS-induced colitis.

Fig S1

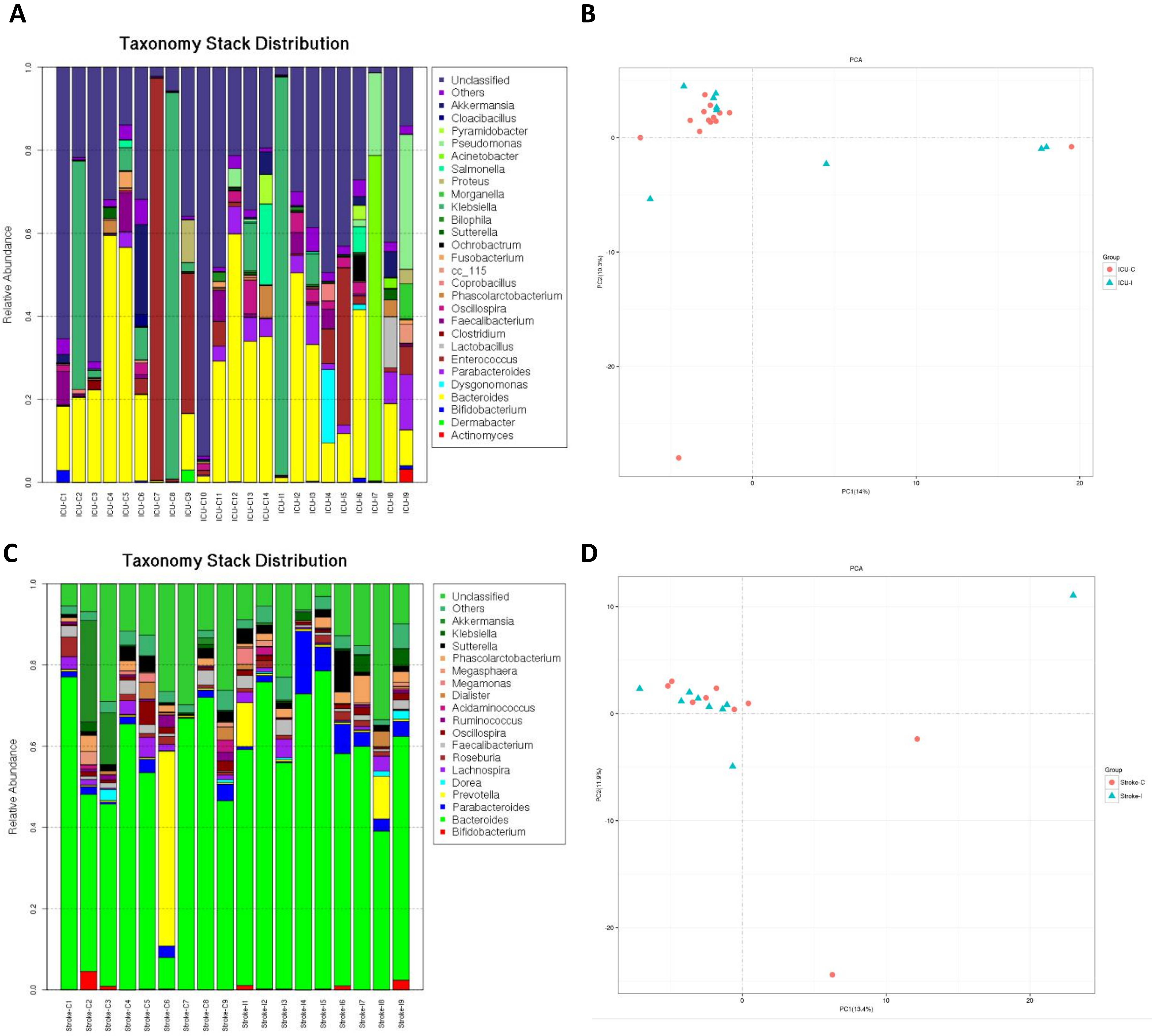
A
Taxonomy Stack Distribution
Relative Abundance
Unclassified
Others
Akkermansia
Cloacibacillus
Pyramidobacter
Pseudomonas
Acinetobacter
Salmonella
Proteus
Morganella
Klebsiella
Bilophila
Sutterella
Ochrobactrum
Fusobacterium
cc_115
Coprobacillus
Phascolarctobacterium
Oscillospira
Faecalibacterium
Clostridium
Lactobacillus
Enterococcus
Parabacteroides
Dysgonomonas
Bacteroides
Bifidobacterium
Dermabacter
Actinomyces
B
PCA
PC1(14%)
Group
ICU-C
ICU-I
C
Taxonomy Stack Distribution
Relative Abundance
Unclassified
Others
Akkermansia
Klebsiella
Sutterella
Phascolarctobacterium
Megasphaera
Megamonas
Dialister
Acidaminococcus
Ruminococcus
Oscillospira
Faecalibacterium
Roseburia
Lachnospira
Dorea
Prevotella
Parabacteroides
Bacteroides
Bifidobacterium
D
PCA
PC1(13.4%)
Group
Stroke-C
Stroke-I

Fig S2

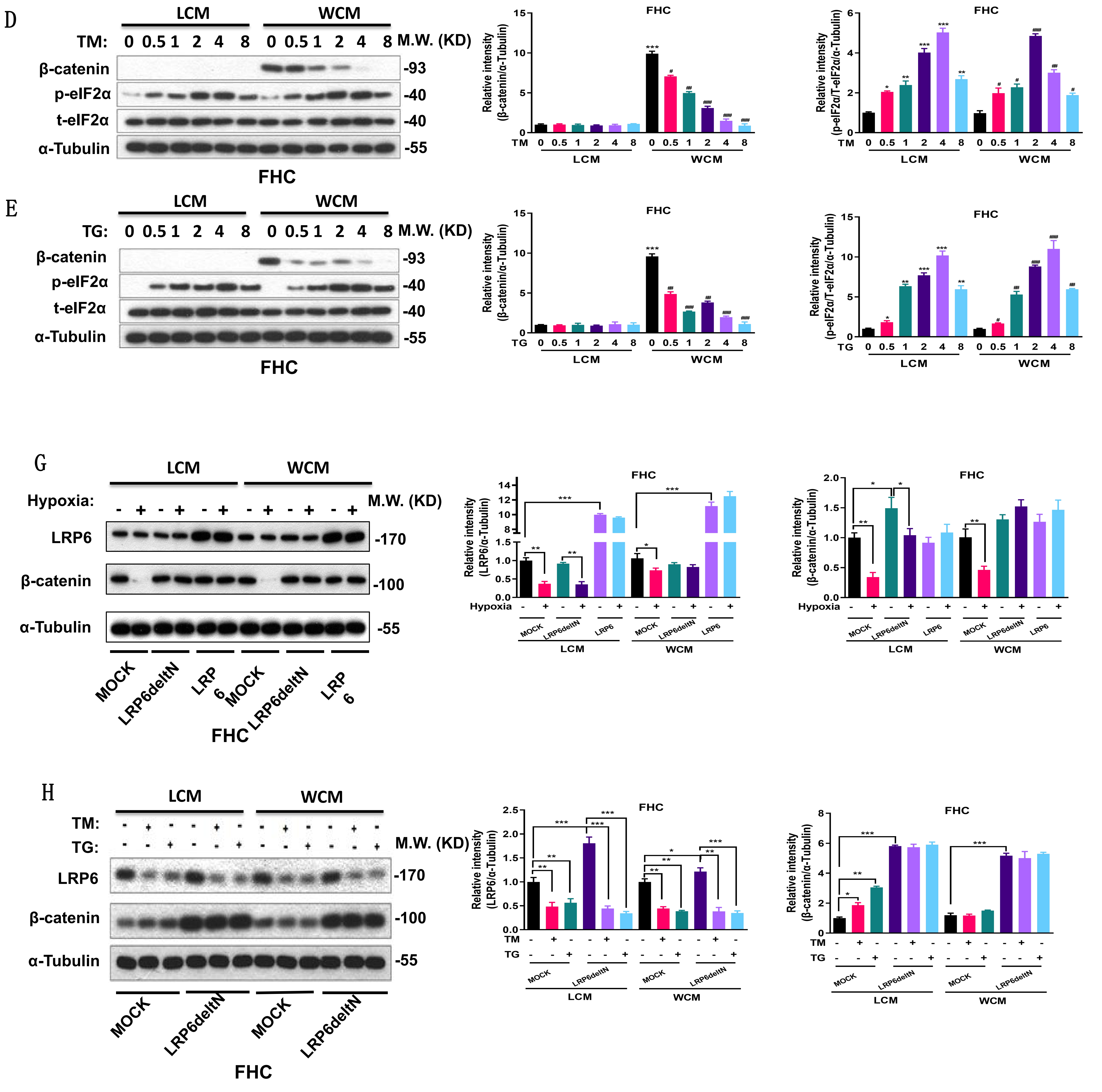
D
LCM
WCM
TM: 0 0.5 1 2 4 8 0 0.5 1 2 4 8 M.W. (KD)
β-catenin -93
p-eIF2α -40
t-eIF2α -40
α-Tubulin -55
FHC
Relative intensity (β-catenin/α-Tubulin)
Relative intensity (p-eIF2α/T-eIF2α/α-Tubulin)
E
TG: 0 0.5 1 2 4 8 0 0.5 1 2 4 8 M.W. (KD)
G
Hypoxia:
LRP6 -170
β-catenin -100
α-Tubulin -55
MOCK
LRP6deltN
LRP6
Relative intensity (LRP6/α-Tubulin)
H
TM:
TG:

Fig S3

A

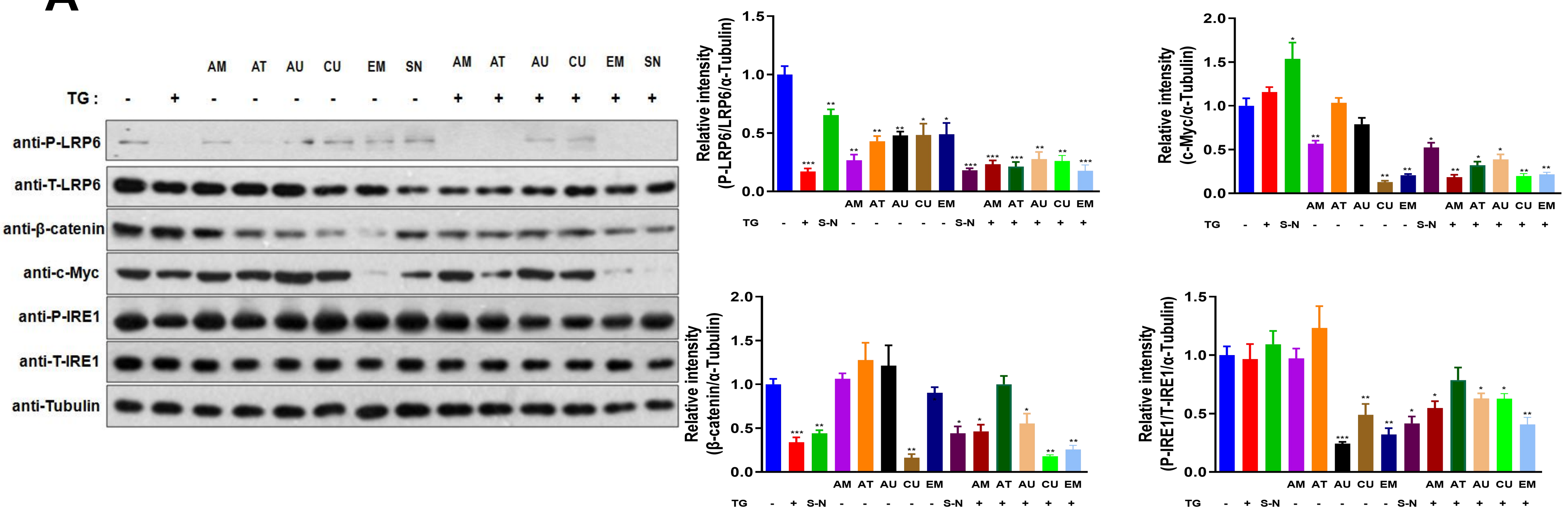
AM AT AU CU EM SN AM AT AU CU EM SN
TG : - + - - - - - - + + + + + +
anti-P-LRP6
anti-T-LRP6
anti-β-catenin
anti-c-Myc
anti-P-IRE1
anti-T-IRE1
anti-Tubulin
Relative intensity (P-LRP6/LRP6/α-Tubulin)
Relative intensity (c-Myc/α-Tubulin)
Relative intensity (β-catenin/α-Tubulin)
Relative intensity (P-IRE1/T-IRE1/α-Tubulin)
TG - + S-N - - - - - S-N + + + + +
AM AT AU CU EM AM AT AU CU EM

G

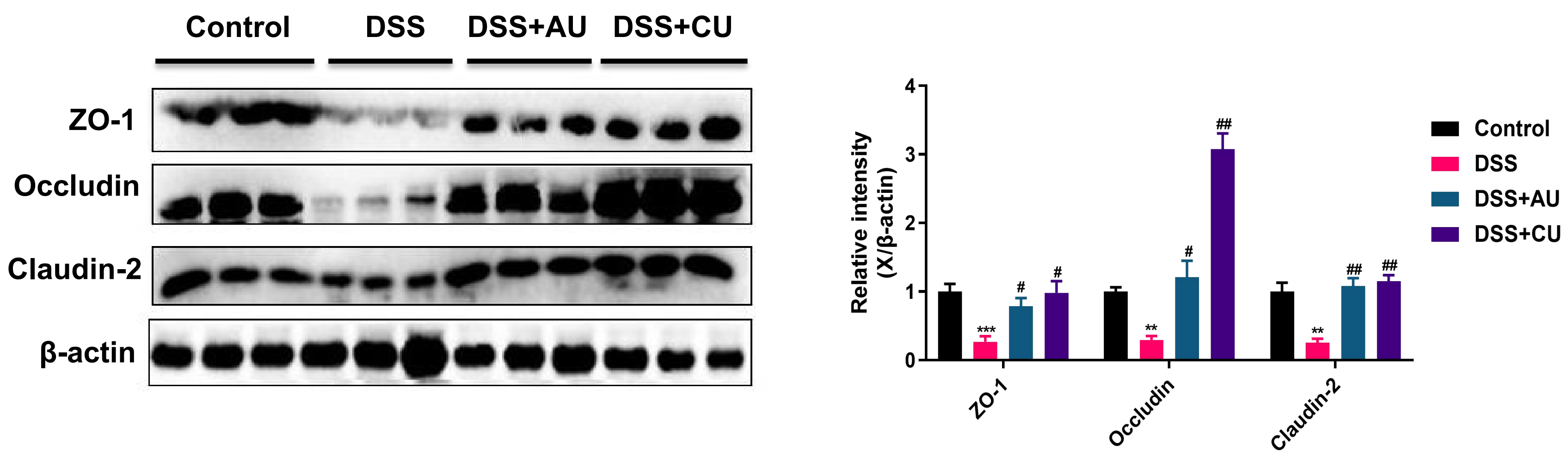
Control
DSS
DSS+AU
DSS+CU
ZO-1
Occludin
Claudin-2
β-actin
Relative intensity (X/β-actin)
Control
DSS
DSS+AU
DSS+CU
ZO-1
Occludin
Claudin-2

H

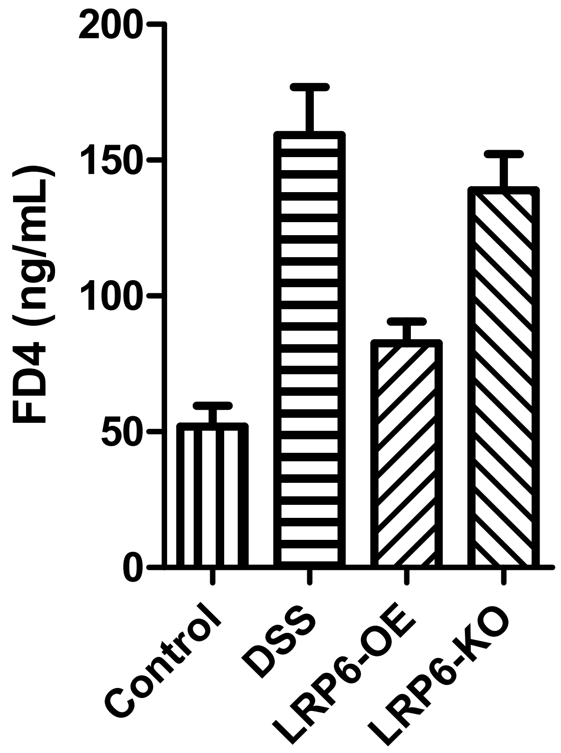
FD4 (ng/mL)
200
150
100
50
0
Control
DSS
LRP6-OE
LRP6-KO

Fig S4

A

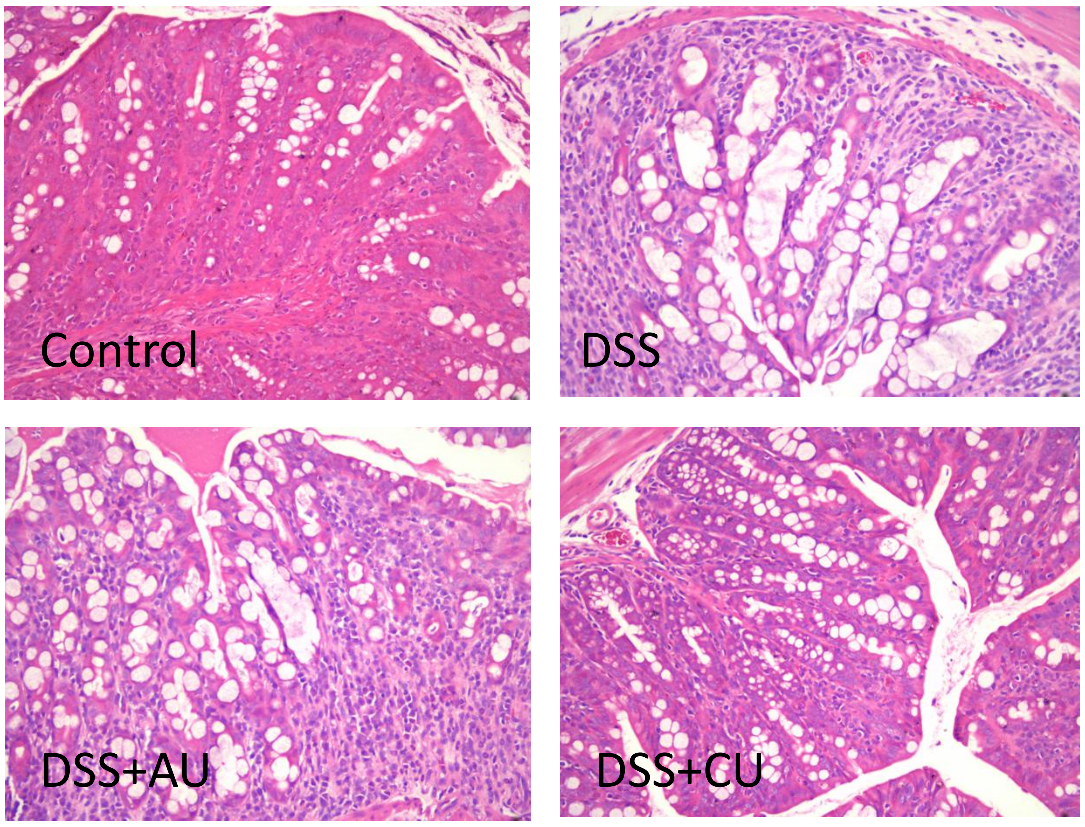

Supplement: Supplementary file 1 — Supplementary figures. [file ijbsv18p4469s1.pdf]
